# Hemistepsin a Induces Apoptosis of Hepatocellular Carcinoma Cells by Downregulating STAT3

**DOI:** 10.3390/ijms22094743

**Published:** 2021-04-29

**Authors:** Il Je Cho, Jae Kwang Kim, Eun Ok Kim, Sang Mi Park, Sang Chan Kim, Sung Hwan Ki, Sae Kwang Ku

**Affiliations:** 1 College of Korean Medicine, Daegu Haany University, Gyeongsan 38610, Korea; skek023@dhu.ac.kr (I.J.C.); kimjk@kiom.re.kr (J.K.K.); keo84@hanmail.net (E.O.K.); miya38@nate.com (S.M.P.); sckim@dhu.ac.kr (S.C.K.); 2 Korean Medicine-Application Center, Korea Institute of Oriental Medicine, Daegu 41062, Korea; 3 College of Pharmacy, Chosun University, Gwangju 61452, Korea; shki@chosun.ac.kr

**Keywords:** apoptosis, *Hemistepta lyrata* (Bunge) Bunge, hemistepsin A (HsA), hepatocellular carcinoma (HCC) cells, oxidative stress, signal transducer and activator of transcription 3 (STAT3), sorafenib

## Abstract

*Hemistepta lyrata* (Bunge) Bunge is a biennial medicinal plant possessing beneficial effects including anti-inflammation, and hemistepsin A (HsA) isolated from *H. lyrata* has been known as a hepatoprotective sesquiterpene lactone. In this report, we explored the cytotoxic effects of *H. lyrata* on hepatocellular carcinoma (HCC) cells and investigated the associated bioactive compounds and their relevant mechanisms. From the viability results of HCC cells treated with various *H. lyrata* extracts, HsA was identified as the major compound contributing to the *H. lyrata*-mediated cytotoxicity. HsA increased expression of cleaved PARP and cells with Sub-G1 phase, Annexin V binding, and TUNEL staining, which imply HsA induces apoptosis. In addition, HsA provoked oxidative stress by decreasing the reduced glutathione/oxidized glutathione ratio and accumulating reactive oxygen species and glutathione-protein adducts. Moreover, HsA inhibited the transactivation of signal transducer and activator of transcription 3 (STAT3) by its dephosphorylation at Y705 and glutathione conjugation. Stable expression of a constitutive active mutant of STAT3 prevented the reduction of cell viability by HsA. Finally, HsA enhanced the sensitivity of sorafenib-mediated cytotoxicity by exaggerating oxidative stress and Y705 dephosphorylation of STAT3. Therefore, HsA will be a promising candidate to induce apoptosis of HCC cells via downregulating STAT3 and sensitizing conventional chemotherapeutic agents.

## 1. Introduction

Repeated and chronic hepatocellular injuries by heterogeneous etiology infiltrate inflammatory cells, which in turn facilitate chromosomal instability by reactive oxygen species (ROS)-mediated mutagenesis, and trigger an abnormal proliferation of parenchymal cells (and progenitor cells) by the sustained production of cytokines. Liver cancer, which is established by a vicious cycle of injury, inflammation, and abnormal proliferation, is the sixth most prevalent cancer and the third leading cause of cancer-related death worldwide [1]. In particular, hepatocellular carcinoma (HCC), which accounts for more than 90% of liver cancers, is more common in Korea and ranks second in cancer mortality [2]. Although surgical resection, liver transplantation, transarterial chemoembolization, radiotherapy, and chemotherapy are accepted interventions for treating HCC [3], treatment options are still limited. For instance, sorafenib, an inhibitor for several receptor tyrosine kinases as well as Raf, is the first line-approved drug for treating advanced HCC [4]. However, sorafenib only extends the survival of patients for three months because of the heterogeneous nature of HCC, drug resistance acquisition, and organ/hematological toxicities [4,5]. Therefore, it is still urgent to develop a more efficacious strategies for the treatment of HCC.

Of diverse signaling molecules, the signal transducer and activator of transcription 3 (STAT3) has been considered as a bona fide oncogenic factor in HCC [6,7]. Canonically, stimulation of cytokines (e.g., interleukin (IL)-6, IL-10 and IL-22) and growth factors (e.g., vascular endothelial growth factor and platelet-derived growth factor) rapidly trans-phosphorylates tyrosine residues of Janus kinases (JAKs) attached in the cytoplasmic domain of the cognate receptors, which serves as a docking site for STAT3 and subsequently phosphorylates tyrosine (Y) 705 of STAT3. The phosphorylated STAT3s dimerize each other and translocate into the nucleus, where they bind to interferon-γ activated sites for transactivating downstream genes [6,8]. Under a normal physiological state, STAT3 activation is usually transient even in the continuous stimulation of cytokines and contributes to protecting the normal hepatocytes from toxic/inflammatory insults [9,10]. However, in the already established HCC, persistent activation of STAT3 accelerates gene transcriptions associated with cell survival, proliferation, invasion, angiogenesis, and cancer stemness [6]. In addition, STAT3 activation blocks immune surveillance in the tumor stroma [11,12] and is associated with acquiring resistance to conventional chemotherapeutic agents [13,14]. Moreover, constitutive phosphorylation of STAT3 in the tumoral tissue is closely correlated with poor prognosis in HCC patients [15,16]. In these regards, small molecules interfering with STAT3 activation have been proposed to treat HCC [6,8]. Furthermore, some bioactive compounds isolated from edible medicinal plants (e.g., sesquiterpene lactone (STL)) show great interest as an alternative resource that regulates STAT3 activity without serious organ toxicity [17,18].

*Hemistepta lyrata* (Bunge) Bunge, a Compositae family, is a biennial herb growing wild in Korea and has been ingested traditionally for managing wounds, fever, hemorrhage, and ulcers [19]. We recently reported that *H. lyrata* crude extract and hemistepsin A (HsA; a guaianolide class of STL isolated from *H. lyrata*) are potential candidates for preventing hepatitis, steatosis, and fibrosis [20,21,22,23]. In addition, HsA has been considered to possess cytotoxic activity against several human cancer cells [24,25,26]. Especially, HsA blocks the Warburg effect by inhibiting pyruvate dehydrogenase kinase, which in turn facilitates the apoptosis of colorectal cancer cells by generating mitochondrial ROS [25]. In addition, HsA attenuates the cell cycle progression of HCC cells and induces senescence through activating AMP-activated protein kinase (AMPK) [24]. Although HsA has been proposed to have therapeutic potential against HCC cells, the relevant cellular mechanisms of HsA have not been fully understood. Furthermore, there were no studies attempting to use HsA as a complementary drug for improving the sensitivity of conventional chemotherapy to HCC.

Therefore, the aim of the present study is to explore the effects of *H. lyrata* extracts on the viability of HCC cells and to elucidate a major cytotoxic compound contained in the parental herb. Moreover, the present study investigates cellular mechanisms associated with HsA-mediated cytotoxicity and further explores the combined effects of HsA with sorafenib in HCC cells.

## 2. Results

### 2.1. HsA in H. lyrata Is a Representative Cytotoxic Compound against HCC Cells

**Figure 1:**
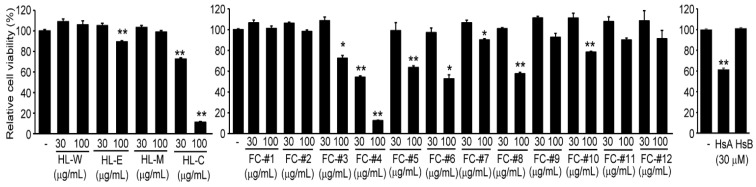
Effects of *H. lyrata* crude extracts or isolated bioactive compounds on the viability of HepG2 cells. After HpeG2 cells were treated with four *H. lyrata* crude extracts (left), twelve fractionated *H. lyrata* chloroform extracts (middle), HsA, or HsB (right) for 24 h, relative cell viability was determined by thiazolyl blue tetrazolium bromide (MTT) assay. ** *p* < 0.01, * *p* < 0.05, significance versus vehicle-treated cells: HL-W, *H. lyrata* water extract; HL-E, *H. lyrata* ethanol extract; HL-M, *H. lyrata* methanol extract; HL-C, *H. lyrata* chloroform extract; FC, fractionated *H. lyrata* chloroform extract.

To explore whether *H. lyrata* has cytotoxic potential against HCC cells, HepG2 cells were treated with 30 and 100 μg/mL of four types of *H. lyrata* crude extracts for 24 h. Compared to vehicle-treated cells, treatment with *H. lyrata* water extract or *H. lyrata* methanol extract did not change the viability of HepG2 cells. On the contrary, *H. lyrata* chloroform extract significantly decreased the viability of HepG2 cells (e.g., 72.62 ± 1.29 and 11.24 ± 0.67% in cells treated with 30 and 100 μg/mL *H. lyrata* chloroform extract, respectively). In addition, 100 μg/mL *H. lyrata* ethanol extract also slightly, but significantly, reduced the viability of HepG2 cells (e.g., 89.42 ± 1.02% of vehicle-treated cells) (Figure 1, left). Since *H. lyrata* chloroform extract showed the most potent cytotoxicity, it was further fractionated based on its solubility in methanol, as previously reported [21]. The magnitude of reduction in cell viability by fractionated *H. lyrata* chloroform extract No. 4 was the most potent among 12 fractionated extracts tested (e.g., 54.19 ± 1.36 and 12.54 ± 0.43% in cells treated with 30 and 100 μg/mL fractionated *H. lyrata* chloroform extract No. 4, respectively) (Figure 1, middle). We previously reported that HsA in fractionated *H. lyrata* chloroform extract No. 4 is a major bioactive compound that inhibits steatosis, hepatitis, and fibrosis [20,21,23]. Therefore, we further explored whether HsA and its congener, hemistepsin B (HsB), can decrease the viability of HepG2 cells. Treatment with 30 μM HsA, but not HsB, significantly reduced the viability of HepG2 cells (e.g., 61.51 ± 1.22% of vehicle-treated cells) (Figure 1, right).

### 2.2. HsA Activates Apoptosis of HCC Cells 

**Figure 2:**
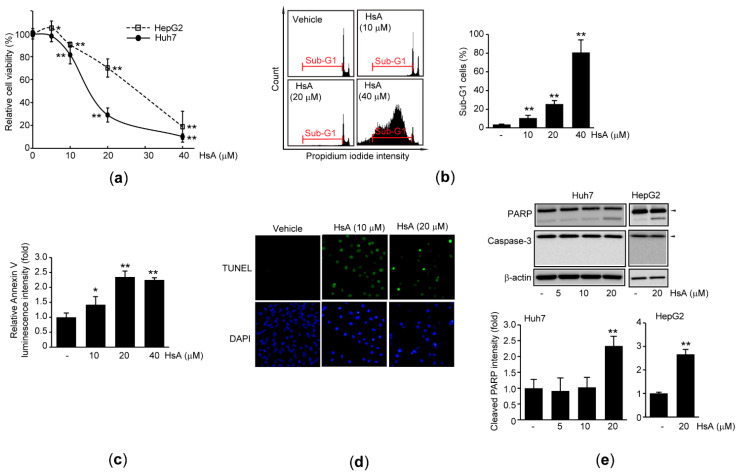
HsA activates the apoptosis of HCC cells. Huh7 (for **a**–**e**) and HepG2 (for **a** and **e**) were exposed to 5–40 μM HsA for 24 (for **b**, **d**, and **e**) or 48 h (for **a**,**c**): (**a**) Relative cell viability of HsA-treated cells was determined by MTT assay; (**b**) Fluorescence intensity of PI-stained cells was monitored using a flow cytometer (left), Sub-G1 cells were expressed as a percentage of the total cell analyzed (right); (**c**) Annexin V binding activity was determined using a luminometer; (**d**) Fragmented DNAs were stained by TUNEL, and nuclei were counter-stained by DAPI; (**e**) Changes of PARP and caspase-3 expression were determined by immunoblotting. Equal protein loading was verified by β-actin immunoblotting. Arrowheads in immunoblot images indicate precursor forms of PARP and caspase-3 (upper). Band intensity of cleaved PARP was quantified by scanning densitometry (lower). ** *p* < 0.01, * *p* < 0.05, significance versus vehicle-treated cells.

To study HsA-mediated cytotoxicity more in-depth, Huh7 and hepG2 cells, two representative human HCC cell lines, were treated with various concentrations of HsA for 48 h. Compared to vehicle-treated cells, significant reductions in relative cell viability were observed in cells treated with 10–40 μM HsA. The inhibitory concentration of 50% (IC_50_) of HsA was 15.27 ± 1.84 and 26.50 ± 6.07 μM in Huh7 cells and HepG2 cells, respectively (Figure 2a). In addition, results from a flow cytometer indicated that HsA significantly increased the percentage of Sub-G1 cells showing low propidium iodide (PI) fluorescence intensity in a concentration-dependent manner (Figure 2b). Moreover, HsA increased the binding of Annexin V as well as the number of cells stained by terminal deoxynucleotidyl transferase dUTP nick end labeling (TUNEL) (Figure 2c,d), which imply that HsA accelerates exposure of phosphatidylserine in the outer leaflet of the plasma membrane and promotes fragmentation of chromosomal DNA. Furthermore, cleavage of Poly (ADP-ribose) polymerase (PARP), but not caspase 3, was significantly increased in both HCC cells treated with HsA (Figure 2e).

### 2.3. Oxidative Stress Triggers HsA-Induced Apoptosis in HCC Cells

**Figure 3:**
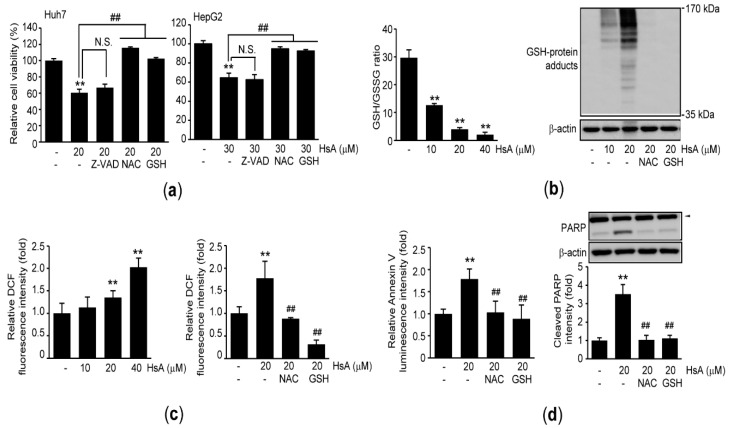
Oxidative stress contributes to promoting HsA-mediated apoptosis in HCC cells. Huh7 (for **a**–**d**) and HepG2 (for **a**) were exposed to 10–40 μM HsA for 6 (for **c**), 24 (for **b** and **d**-right), or 48 h (for **a** and **d**-left). Z-VAD (20 μM), NAC (1 mM), or GSH (2 mM) was pre-incubated for 1 h prior to HsA treatment: (**a**) MTT assay; (**b**) The GSH/GSSG ratio was calculated after quantifying reduced and oxidized glutathione in HsA-treated cells (left). GSH-protein adducts ranging from 35 to 170 kDa were determined by GSH immunoblotting (right); (**c**) ROS was monitored after incubating cells with 2′,7′-dichlorofluorescein diacetate (DCFH-DA); (**d**) Annexin V binding activity (left) and expression level of cleaved PARP (right). Arrowhead in immunoblot image indicates PARP precursor (upper right). Band intensity of cleaved PARP was quantified by densitometry (lower right). ** *p* < 0.01, significance versus vehicle-treated cells; ^##^ *p* < 0.01, significance among HsA-treated cells; N.S., not significant; DCF, dichlorofluorescein.

Dysregulated oxidative stress has been known as one of the major executors to provoke apoptosis. To explore cellular mechanisms for HsA-mediated apoptosis in HCC cells, HCC cells were pre-incubated with pan-caspase inhibitor (e.g., Z-VAD-FMK (Z-VAD)) or antioxidants (e.g., N-acetylcysteine (NAC)) and reduced glutathione (GSH)) for 1 h prior to HsA treatment. In parallel with results from caspase-3 immunoblotting (Figure 2e), pre-incubating HCC cells with Z-VAD did not change the decrease in cell viability by HsA. However, pretreatment with NAC or GSH significantly prevented the HsA-mediated reduction of cell viability (Figure 3a). In addition, HsA significantly decreased the ratio of GSH/oxidized glutathione (GSSG) and accumulated GSH-protein adducts as well as cellular ROS (Figure 3b,c and Figure S1), which suggest that HsA provokes oxidative stress in HCC cells. However, the accumulation of GSH-protein adducts and ROS induced by HsA was completely abolished in the presence of NAC or GSH (Figure 3b,c and Figure S1). Moreover, pretreatment with the antioxidants significantly reduced the Annexin V binding and PARP cleavage in response to HsA (Figure 3d).

### 2.4. HsA Inhibits STAT3 in HCC Cells

**Figure 4:**
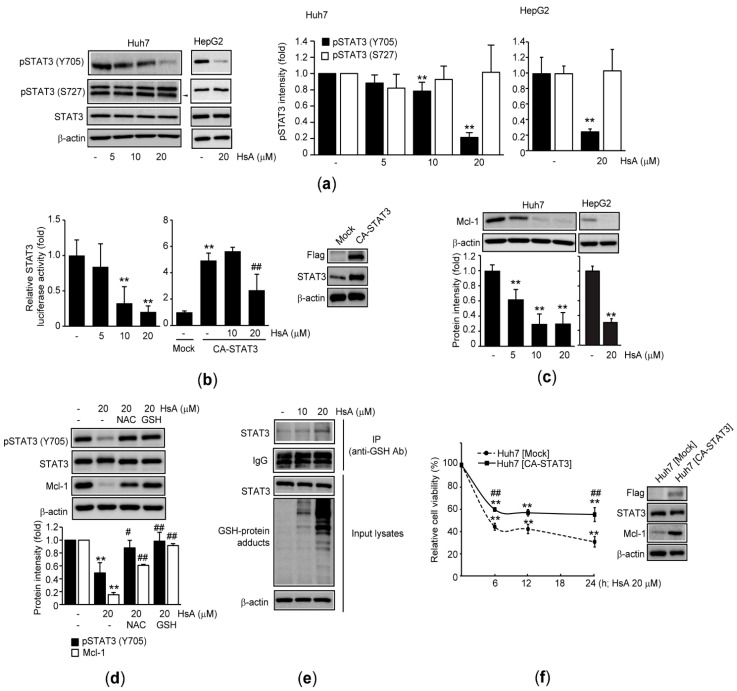
HsA inhibits STAT3 in HCC cells. Huh7 (for **a**–**e**), HepG2 (for **a** and **c**), and recombinant Huh7 cells (for **f**) were exposed to 5–20 μM HsA for 18 (for **b**), 24 (for **a**, **c**–**e**), or 6–24 h (for **f**): (**a**) Immunoblot analysis for phosphorylated STAT3 in HsA-treated HCC cells. Arrowhead in immunoblot image indicates a non-specific band (left). Band intensity of phosphorylated STAT3 in Huh7 (middle) and HepG2 (right) was quantified by densitometry; (**b**) Reporter gene assay. Huh7 cells were transiently transfected with pGL4.47[luc2P/SIE/Hygro] alone (left) or in combination with CA-STAT3. Equal amount of pCDNA3.2/V5-DEST was used as mock-transfection (middle and right). Expression of CA-STAT3 was verified by Flag immunoblotting (right); (**c**) Mcl-1 expression in HsA-treated HCC cells (upper). Mcl-1 levels were quantified by densitometry (lower); (**d**) Effect of antioxidants on HsA-dependent STAT3 inhibition. NAC (1 mM)-, or GSH (2 mM)-pretreated Huh7 cells were exposed to HsA (upper). Levels of phosphorylated STAT3 at Y705 and Mcl-1 expression were quantified by densitometry (lower); (**e**) Proteins obtained from HsA-treated Huh7 cells were immunoprecipitated using an anti-GSH antibody, followed by STAT3 immunoblotting. 30 μg of proteins was used as input control lysates; (**f**) MTT assay was conducted after recombinant Huh7 cells were exposed to HsA (left). Stable expression of CA-STAT3 was verified by Flag immunoblotting (right). ** *p* < 0.01, significance versus vehicle-treated cells (for **a**,**b**-left,**c**,**d**, **f**) or mock-transfected cells (for **b**-middle); ^##^ *p* < 0.01, ^#^ *p* < 0.05, significance among CA-STAT3-transfected cells (for **b**-middle), significance among HsA-treated cells (for **d**), significance between Huh7 [Mock] and Huh7 [CA-STAT3] (for **f**-left); pSTAT3, phosphorylated STAT3; IgG, immunoglobulin G; IP, immunoprecipitation; Huh7 [Mock], recombinant Huh7 cells which stably transfected with pCDNA3.2/V5-DEST; Huh7 [CA-STAT3], recombinant Huh7 cells which stably transfected with CA-STAT3.

As aberrant activation of STAT3 signaling pathways contributes to many diseases including cancer [6,8], we further explored the role of HsA on STAT3 activation in HCC cells. HsA treatment significantly decreased the Y705 phosphorylation of STAT3 in both Huh7 and HepG2 cells, while HsA did not alter the expression of STAT3 as well as S727 phosphorylation of STAT3 (Figure 4a). In addition, results from the reporter gene assay indicated that HsA significantly inhibited the luciferase activity by basal STAT3 transactivation (Figure 4b, left) and by ectopic expression of a constitutive active mutant of STAT3 (CA-STAT3) (Figure 4b, middle and right). Moreover, HsA treatment decreased the protein and mRNA level of myeloid cell leukemia-1 (Mcl-1), a downstream target of STAT3 activation (Figure 4c and Figure S2).

To investigate the role of STAT3 downregulation on oxidative stress-mediated apoptosis, Y705 phosphorylation of STAT3 and Mcl-1 expression were observed in antioxidants-pretreated Huh7 cells. Pretreatment with NAC or GSH significantly blocked HsA-mediated decreases in the levels of Y705 phosphorylation of STAT3 and Mcl-1 expression (Figure 4d). In addition, the immunoprecipitation–immunoblot analysis showed that HsA increased GSH-conjugated STAT3 (Figure 4e). Furthermore, a stable expression of CA-STAT3 in Huh7 cells partly, but significantly, alleviated HsA-mediated cytotoxicity, as compared to recombinant Huh7 cells expressing mock plasmid (Figure 4f), suggesting that HsA exhibits cytotoxicity of HCC cells via the downregulation of STAT3.

### 2.5. HsA Sensitizes Sorafenib-Mediated Cytotoxicity in HCC Cells

**Figure 5:**
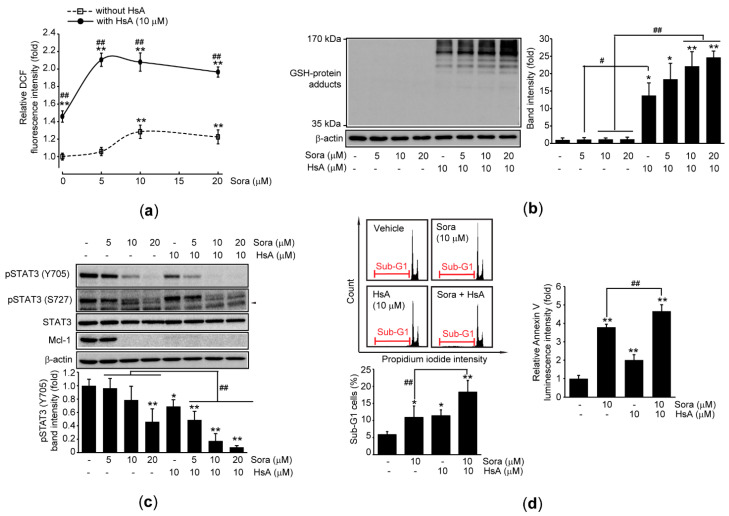
HsA sensitizes sorafenib-mediated cytotoxicity in HCC cells. Huh7 cells were treated with 5–20 μM sorafenib in the presence 10 μM HsA for 6 (for **a**), 24 (for **b, c,** and **d**-left), or 48 h (for **d**-right): (**a**) Effect of HsA on sorafenib-mediated ROS production was monitored by reacting the cells with DCFH-DA; (**b**) Immunoblot analysis using a GSH antibody (left). Intensity of GSH-protein adducts ranging from 35 to 170 kDa was quantified by densitometry (right); (**c**) Immunoblot analysis for phosphorylated STAT3 and Mcl-1 expression. Arrowhead in immunoblot image indicates a non-specific band (upper). Band intensity of Y705 phosphorylation of STAT3 was quantified by densitometry (lower); (**d**) Fluorescence intensity of PI-stained cells (upper left) and Sub-G1 cells (lower left) was analyzed using a flow cytometer. Annexin V binding activity was determined using a luminometer (right). ** *p* < 0.01, * *p* < 0.05, significance versus vehicle-treated cells; ^##^ *p* < 0.01, ^#^ *p* < 0.05, significance between sorafenib and sorafenib + HsA; Sora, sorafenib.

Finally, we explored the effects of HsA on sorafenib-mediated cytotoxicity in HCC cells. Intracellular ROS production was slightly, but significantly, increased when Huh7 cells were exposed to 10 and 20 μM sorafenib for 6 h. As compared to sorafenib alone treated cells, co-treatment with HsA (10 μM) in the presence of sorafenib potentiated ROS production (Figure 5a). Although GSH-protein adducts could not be detected in sorafenib alone treated cells, sorafenib significantly escalated HsA-mediated increases in GSH-protein adducts (Figure 5b). In addition, HsA further reduced the decrease in Y705 phosphorylation of STAT3 in response to sorafenib, while combination with HsA did not affect the reduction of phosphorylated STAT3 at S727 by sorafenib (Figure 5c). Moreover, the combination of HsA and sorafenib significantly increased the percentage of Sub-G1 cells and Annexin V binding activity compared to the treatment with sorafenib alone (Figure 5d).

## 3. Discussion

Throughout thiazolyl blue tetrazolium bromide (MTT) assays using *H. lyrata* crude extracts and fractionated extracts, present results imply that HsA is a responsible STL for *H. lyrata*-mediated cytotoxicity against HCC cells. On the contrary, we and others previously suggested that treatment with sublethal concentrations of HsA (e.g., up to 30 μM HsA in RAW 264.7 cells and 10 μM HsA in HaCaT cells) contributes to reducing ROS production and protecting the cells from oxidative stress through activation of nuclear factor erythroid 2-related factor 2 (Nrf2) [23,27]. However, in the present study, we showed that a similar range of HsA, which was less cytotoxic in the previous studies [23,27], could decrease the viability of HCC cells. In addition, our supplementary reporter gene assay using a luciferase construct harboring four copies of antioxidant response element showed that Nrf2 transactivation peaked with the 5 μM HsA treatment in Huh7 cells (i.e., 5 μM of HsA did not affect the viability of Huh7 cells, as shown in Figure 2a). However, 10–40 μM of HsA decreased Nrf2 transactivation as compared to 5 μM HsA treatment (Figure S3). As the balance between an antioxidant defense system and oxidative stress is a critical factor for determining the fate of cells [28], the decrease in luciferase activity by treatment with high concentrations of HsA may be due to the severe cytotoxicity of HsA beyond its Nrf2-dependent adaptative capacity. Therefore, in the present study, we focused more on HsA-mediated cytotoxic mechanisms than its dependent cytoprotective mechanisms (e.g., Nrf2 activation).

In the present study, we showed that HsA significantly increased annexin V binding to the plasma membrane. Results from PI and TUNEL staining indicate that HsA induces chromosomal fragmentation. Although these phenotypic changes are representative characteristics of apoptosis, we did not detect cleavage of caspase-3 in HsA-treated HCC cells. Moreover, pretreatment with Z-VAD did not alter the HsA-mediated decrease in cell viability, while 20 μM Z-VAD is a sufficient concentration to inhibit caspases in both Huh7 and HepG2 cells [29,30]. Permeabilization of the mitochondrial outer membrane by ROS can release mitochondrial proteins that reside in intermembrane space. Of diverse mitochondrial proteins, apoptosis-inducing factor and endonuclease G are able to translocate into the nucleus and execute apoptotic cell death without activating caspases [31]. Furthermore, PARP is a substrate for caspases as well as other serine proteases (e.g., calpain, cathepsin, granzyme, and matrix metalloproteinase) that are activated in the process of other types of cell death [32]. Thus, our present results raise the possibility that HsA may induce caspase-independent apoptotic cell death.

The intracellular GSH level is generally elevated to adapt from extraordinary energetic metabolism in cancer cells [33], and α-methylene-γ-lactone in STL is a strong electrophile group that is capable of reacting with the cellular sulfhydryl group via Michael addition reaction [17,34]. Thus, disruption of redox homeostasis by STL (e.g., GSH depletion) has been proposed as a representative cellular mechanism to promote cytotoxicity against cancer cells [17,18]. Similar to other STLs, HsA also decreased GSH/GSSG ratio and accumulated ROS in HCC cells. In addition, it has also been shown for the first time that HsA can increase GSH-protein adducts in HCC cells. Moreover, pretreatment with antioxidants (e.g., NAC or GSH) completely prevented HsA-mediated viability reduction, Annexin V binding, and PARP cleavage, suggesting HsA activates apoptosis of HCC cells via inducing oxidative stress. Furthermore, present results showing HsA-mediated decreases in Y705 phosphorylation of STAT3 and Mcl-1 expression were attenuated in the presence of antioxidants imply that oxidative stress in response to HsA triggers inactivation of STAT3 in HCC cells.

It has been reported that JAKs and Src family kinases phosphorylate tyrosine residue in the C-terminal transactivation domain of STAT3 (e.g., Y705) [17]. As the tyrosine phosphorylation in the transactivation domain is essential for STAT3 dimerization and interaction with transcriptional complex [17,35], it is tightly controlled by several negative regulators. For instance, the suppressor of cytokine signaling proteins can inhibit STAT3 phosphorylation by alternative binding to the phosphorylated receptor/adaptor instead of STAT3. In addition, the suppressor of cytokine signaling protein also recruits the ubiquitin-dependent proteasome system for degrading targets [36]. Moreover, protein tyrosine phosphatases directly dephosphorylate the STAT3 and JAKs [37,38]. In the present study, we showed that Y705 phosphorylation, but not S727, of STAT3 was specifically decreased in HsA-treated HCC cells. In addition, results from a reporter gene assay and Mcl-1 expression indicated that HsA could downregulate the transactivation of STAT3. Moreover, ectopic expression of CA-STAT3 partly, but significantly, prevented the reduction of cell viability against HsA. Although present results provide evidence that HsA reduces the viability of HCC cells via inhibiting Y705 phosphorylation and transactivation of STAT3, signaling molecules responsible for HsA-dependent Y705 dephosphorylation of STAT3 need to be further clarified in the future.

In addition to phosphorylation, other covalent modifications also affect the activity of STAT3. Especially, GSH conjugation of STAT3 has been proposed as one of the major pharmacological targets of STLs for anticancer activity [17,18]. Like other STLs (e.g., alantolactone, cynaropicrin, dehydrocostu lactone, and costunolide) [39,40,41], in the present study, we also showed that HsA increased GSH conjugation of STAT3 in HCC cells. Glutathione conjugation of STAT3 has been reported to suppress Y705 phosphorylation, nuclear translocation, and target gene expression [42]. In addition, C328 and C542 of STAT3 have been identified as major cysteine residues responsible for GSH conjugation [43]. Studies from circular dichroism spectra using glutathionylated STAT3 and in silico 3D structural prediction have suggested that the conformational alterations by GSH conjugation probably interfere with JAK’s recognition against the tyrosine residue of STAT3, which leads to inhibit transactivation of STAT3 [17,43]. As the present results showed that HsA accumulated many GSH-protein adducts in HCC cells, HsA may exhibit anticancer activity by glutathionylation of STAT3 as well as other redox-sensitive proteins, which are essential for the survival of HCC cells.

Recently, Baek and colleagues have reported that AMPK activation by treatment with HsA contributes to impairing mitochondrial membrane potential and inducing senescence of Huh7 cells [24]. To test the possibility that AMPK activation plays a critical role in HsA-mediated reduction of the viability of HCC cells, Huh7 and HepG2 cells were preincubated with 10 μM compound C (a chemical inhibitor of AMPK) for 1 h and subsequently exposed to 20 or 30 μM HsA for 48 h. Our supplementary results from MTT assay showed that pretreatment with compound C rather reduced the HsA-mediated decrease in cell viability (Figure S4), suggesting AMPK may not be a major signaling molecule that triggers HsA-mediated cytotoxicity of HCC cells, at least in our experimental condition. On the other hand, accumulated evidence suggests that many STLs exhibit beneficial effects via perturbating nuclear factor-κB (NF-κB) signaling pathways [18,34]. Previously, we also reported that HsA prevents hepatic inflammation and promoted apoptosis of activated hepatic stellate cells through inhibiting NF-κB and Akt signaling pathways [21,23]. Dysregulated activation of Akt by hepatocyte-specific PTEN deficiency has been reported to cause HCC [44], and NF-κB has been considered another important signaling molecule linking hepatic inflammation and HCC [45]. In these regards, we further investigated the effects of HsA on the phosphorylation of Akt and NF-κB in HCC cells. Our additional results indicated that HsA treatment significantly decreased the phosphorylation of Akt and p65 (a representative subunit comprising NF-κB) in Huh7 cells (Figure S5a,b). Therefore, reduction of Akt and NF-κB phosphorylation, as well as inhibition of STAT3 activation, may cooperatively contribute to the decrease in cell viability by HsA, while it is necessary to further study signaling networks between Akt, NF-κB and STAT3 in HCC cells.

In the present study, we showed that HsA could sensitize sorafenib-mediated cytotoxicity by facilitating the accumulation of ROS production and GSH-protein adducts. Although HsA no longer altered the downregulation of S727 phosphorylation of STAT3 by sorafenib, it further decreased the sorafenib-dependent Y705 dephosphorylation of STAT3. More interestingly, it has been also reported that activation of Akt and NF-κB is closely associated with acquiring resistance to sorafenib [46,47]. Consistent with previous reports, our additional immunoblot results showed that sorafenib (5 and 10 μM, 24 h) significantly increased the phosphorylation of Akt and p65 in Huh7 cells (Figure S5c,d). However, sorafenib-induced phosphorylation of Akt and p65 was significantly inhibited in combined treatment with HsA (Figure S5c,d), suggesting HsA may have the potential for inhibiting the viability of HCC cells that have already acquired resistance to sorafenib.

Although the present study provides evidence that HsA has a therapeutic potential to HCC cells in vitro, several limitations should be further overcome to consolidate and expand present findings. In addition to HepG2 (wild type p53) and Huh7 (p53 mutant) cells, it was reported that the viability of SNU-475 (p53 mutant and hepatitis B virus-positive) and SK-Hep-1 (B-Raf and CDKN2A mutants) was also reduced by HsA treatment [24]. However, it is necessary to further investigate the effects of HsA on normal human hepatocytes as well as other HCC cells with different genetic backgrounds. Moreover, diverse in vivo experiments including validation of therapeutic efficacy of HsA (with sorafenib) in xenograft animals, pharmacokinetics and tissue distribution study to predict dosage of HsA, and toxicological properties of HsA are also required for developing HsA as a complementary candidate against HCC in human.

## 4. Materials and Methods

### 4.1. Reagents

*H. lyrata* crude extracts using four different solvents, twelve fractionated *H. lyrata* chloroform extracts, HsA, and HsB were prepared, as described previously [21]. Anti-PARP, anti-caspase-3, anti-phosphorylated STAT3 (Y705), anti-phosphorylated STAT3 (S727), anti-STAT3, anti-Mcl-1, and horseradish peroxidase-conjugated secondary antibodies were obtained from Cell Signaling Technology (Beverly, MA, USA). An antibody against GSH and protein A/G magnetic bead were supplied by Thermo Fisher Scientific (Rockford, IL, USA). Z-VAD and sorafenib tosylate were provided by Calbiochem (San Diego, CA, USA) and Santa Cruz Biotechnology (Santa Cruz, CA, USA), respectively. MTT, antibodies for β-actin and Flag, NAC, GSH, 2′,7′-dichlorofluorescein diacetate (DCFH-DA), and other reagents were purchased from Sigma-Aldrich (St. Louis, MO, USA).

### 4.2. Cell Culture and Drug Treatment

Huh7 and HepG2 cells derived from human hepatocellular carcinoma were obtained from a Korean Cell Line Bank (Seoul, Korea) and American Type Culture Collection (Manassas, VA, USA), respectively. The cells were maintained under Dulbecco’s modified Eagle medium (DMEM; HyClone Laboratories, Logan, UT, USA) with 10% fetal bovine serum (FBS; HyClone Laboratories) and 1% Antibiotic-Antimycotic solution (Thermo Fisher Scientific). When HepG2 or Huh7 cells were grown to about 80% confluency in an appropriate multi-well plate, the medium in each well was exchanged with a fresh medium containing 10% FBS. The cells were then treated with *H. lyrata* crude extract (30 or 100 μg/mL each), fractionated *H. lyrata* chloroform extract (30 or 100 μg/mL each), HsB (30 μM), or HsA (5–40 μM) for the indicated time periods. Equal volumes of dimethyl sulfoxide (DMSO) were treated as a vehicle. In some experiments, cells were pre-exposed to Z-VAD (20 μM), NAC (1 mM), or GSH (2 mM) 1 h before HsA treatment. Sorafenib (5–20 μM) and HsA (10 μM) were simultaneously exposed to cells in the case of a combination of the two drugs.

### 4.3. MTT Assay

Treated cells were additionally incubated with MTT (0.5 mg/mL) for 2 h. After discarding the medium, formazan crystals were dissolved in DMSO and the absorbance was measured at 570 nm using a Synergy HTX Multi-Mode Plate Reader (BioTek, Winooski, VT, USA). Relative cell viability was expressed as a percentage of the optical intensity in the drug-treated cells relative to the vehicle-treated cells. IC_50_ was calculated using GraphPad Prism (version 9.0.2 for Windows; San Diego, CA, USA).

### 4.4. Measurement of Cell Population in Sub-G1 Phase

Treated cells were detached from 6-well plates by Trypsin-EDTA (HyClone Laboratories) and subsequently stained with PI using a BD Cycletest^TM^ Plus DNA kit (BD Biosciences, San Jose, CA, USA) according to the manufacturer’s instructions. The percentage of cells exhibiting low PI intensity (Sub-G1 phase) was analyzed using an Accuri^TM^ C6 Plus flow cytometer (BD Biosciences). 10,000 cells were recorded in each experiment.

### 4.5. Apoptosis Assay

Phosphatidylserine in the outer leaflet of the plasma membrane was detected using Realtime-Glo^TM^ Annexin V Apoptosis Assay (Promega, Madison, WI, USA). In addition, fragmented DNAs were stained using DeadEnd^TM^ Fluorometric TUNEL System (Promega), as described previously [21]. After the stained cells were mounted with VECTASHIELD^®^ Antifade Mounting Medium containing DAPI (Vector Laboratories, Burlingame, CA, USA), the cells were observed under an Eclipse Ti-U fluorescence microscope (Nikon, Kanagawa, Japan).

### 4.6. Protein Extraction, Immunoprecipitation, and Immunoblot Analysis

Protein extraction using a radioimmunoprecipitation buffer, protein quantification, sodium dodecyl sulfate-polyacrylamide gel electrophoresis, and immunoblot analysis was conducted as described previously [21]. In the case of immunoprecipitation, proteins (about 1 mg) incubated overnight with an anti-GSH antibody were immunoprecipitated for 2 h by adding a protein A/G magnetic bead. An immune complex was then solubilized by boiling in 2× Laemmli sample buffer. After detecting immunoreactive bands of interest using an Imager 600 (GE Healthcare Life Sciences, Little Chalfont, UK), the band intensity was quantified by ImageJ (https://imagej.nih.gov/ij; accessed on 12 June 2020). The expression level of specific protein was normalized by that of β-actin.

### 4.7. Measurement of Glutathione

Levels of GSH and GSSG were measured using GSH/GSSG-Glo^TM^ Assay (Promega), according to the manufacturer’s instructions.

### 4.8. Measurement of Cellular ROS

Huh7 cells were treated with drugs in the presence of 10 μM DCFH-DA for 6 h, and fluorescence intensity of 2′,7′-dichlorofluorescein was measured at 485 nm (excitation) and 530 nm (emission) using an Infinite 200 Pro microplate reader (Tecan, Männedorf, Switzerland).

### 4.9. Plasmids and Reporter Gene Assay

The Stat3-C Flag pRc/CMV (CA-STAT3) was a gift from Jim Darnell (Addgene plasmid #8722) [48], and pCDNA3.2/V5-DEST was from Thermo Fisher Scientific. pGL4.47[*luc2P*/SIE/Hygro] (a firefly luciferase reporter plasmid under the control of five copies of STAT3 responsive element) and pRL-SV (a *Renilla* luciferase reporter plasmid under the control of SV40 promoter) were provided by Promega. Huh7 cells in Opti-MEM medium (Thermo Fisher Scientific) were transiently transfected with pGL4.47[*luc2P*/SIE/Hygro] (300 ng) and pRL-SV (30 ng) for 6 h using a FuGENE^®^ HD Transfection Reagent (Promega). After replacing the transfection medium with the growth medium containing 10% FBS, the cells were treated with 5–20 μM HsA for 18 h. In some experiments, Huh7 cells were transfected with 300 ng of CA-STAT3 in conjunction with reporter plasmids for 12 h, and then exposed to HsA. The same amount of pCDNA3.2/V5-DEST was used for mock transfection. Luciferase activities in the cell lysates were determined using a Dual-Luciferase^®^ Reporter Assay System (Promega), and luminescence intensity of firefly luciferase was normalized by that of *Renilla* luciferase.

### 4.10. Generation of Recombinant Cells Expressing CA-STAT3

Huh7 cells that had been transfected with either CA-STAT3 or pCDNA3.2/V5-DEST were subsequently maintained in a growth medium containing 500 μg/mL of G418 disulfate (Roche Molecular Biochemicals, Indianapolis, IN, USA). Isolated resistance colonies were pooled and used as recombinant cells. Ectopic expression of CA-STAT3 was verified by Flag immunoblotting.

### 4.11. Statistical Analysis

Numerical results were expressed as the mean ± standard deviation of at least three separate experiments. Means between two groups were compared by Student’s t-test. According to the results from Levene’s test, a one-way analysis of variance or Welch’s test was conducted to compare means among experimental groups. Tukey’s honestly significant difference (for equal variance samples) or Dunnett’s T3 test (for non-equal variance samples) were used as post hoc analysis. *p* < 0.05 was considered as a statistical significance of difference.

## 5. Conclusions

The present results provide evidence that HsA isolated from *H. lyrata* is a cytotoxic STL against HCC cells through inducing oxidative stress and downregulating STAT3 activity. Therefore, HsA will be a promising bioactive compound that exerts cytotoxicity against HCC cells and improves the sensitivity of conventional chemotherapeutic agents.

## Data Availability

The data presented in this study are available on request from the corresponding author.

## Supplementary Materials

The following are available online at https://www.mdpi.com/article/10.3390/ijms22094743/s1, Figure S1: Effect of antioxidants on HsA-induced GSH-protein adducts, Figure S2: qPCR analysis, Figure S3: Nrf2 transactivation by HsA, Figure S4: effect of compound C on HsA-mediated toxicity, Figure S5: Effect of HsA on Akt and NF-κB phosphorylation in HCC cells.
