# Supplementary material for: Hemistepsin a Induces Apoptosis of Hepatocellular Carcinoma Cells by Downregulating STAT3
Source: Int J Mol Sci. 2021 Apr 29;22(9):4743. doi: 10.3390/ijms22094743 (PMC8125382; doi:10.3390/ijms22094743)
Supplement: Supplementary file 1 [file ijms-22-04743-s001.zip › ijms-1191831-supplementary.pdf]

International Journal of
Molecular Sciences

MDPI

Supplementary Information

Title: Hemistepsin A Induces Apoptosis of Hepatocellular Carcinoma Cells by Downregulating STAT3

Author's name: Il Je Cho[1,†], Jae Kwang Kim[1,2,†], Eun Ok Kim[1], Sang Mi Park[1], Sang Chan Kim[1], Sung Hwan Ki[3], Sae Kwang Ku[1,*]

Affiliation: [1]College of Korean Medicine, Daegu Haany University, Gyeongsan, Gyeongsangbuk-do 38610, Korea; [2]Korean Medicine-Application Center, Korea Institute of Oriental Medicine, Daegu 41062, Korea; [3]College of Pharmacy, Chosun University, Gwangju 61452, Korea

[*]**Corresponding Author's Information:** Department of Anatomy and Histology, College of Korean Medicine, Daegu Haany University, Gyeongsan, Gyeongsangbuk-do 38610, Korea. Tel: +82-53-819-1549, E-mail: gucci200@dhu.ac.kr

[†]I.J.C. and J.K.K. contributed equally to this work.

Supplementary Materials

pGL4.37[*luc2P*/ARE/Hygro] and compound C were supplied by Promega (Madison, WI, USA) and Calbiochem (San Diego, CA, USA), respectively. Antibodies against phosphorylated Akt (S473), Akt, and phosphorylated p65 (S536) were obtained from Cell Signaling Technology (Beverly, MA, USA). Anti-p65 antibody was provided by Santa Cruz Biotechnology (Santa Cruz, CA, USA).

Quantitative Polymerase Chain Reaction (qPCR)

Total RNA was isolated using a Tri-solution (Bioscience Technology, Daegu, Korea) and subsequently reverse-transcribed for obtaining cDNA. qPCR was carried out with CFX96™ Real-Time System (Bio-Rad, Hercules, CA, USA) using TB Green Premix Ex Taq™ (Takara, Shiga, Japan). The following oligonucleotides were used to amplify specific genes: human Mcl-1, 5’-GGACATCAAAAACGAAGACG-3’ (forward), 5’-GCAGCTTTCTTGGTTTATGG-3’ (backward); human glyceraldehyde 3-phosphate dehydrogenase 5’-GAAGGTGAAGGTCGGAGTC-3’ (forward), 5’-GAAGATGGTGATGGGATTTC-3’ (backward). The relative expression of Mcl-1 was calculated using glyceraldehyde 3-phosphate dehydrogenase as a house keeping gene [1,2].

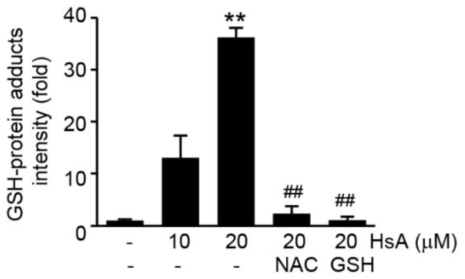

Figure S1. Effect of antioxidants on HsA-induced GSH-protein adducts. Intensity of GSH-protein adducts ranging from 35 to 170 kDa was quantified by densitometry. $^{**}p < 0.01$, significance versus vehicle-treated cells; $^{\#\#}p < 0.01$, significance between HsA-treated cells.

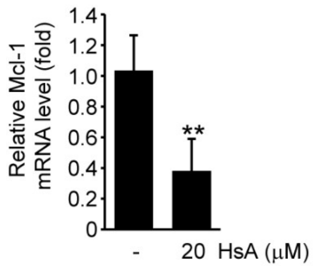

Figure S2. qPCR analysis. Level of Mcl-1 mRNA was quantified from the Huh7 cells treated with 20 μM HsA for 24 h. ** $p < 0.01$, significance versus vehicle-treated cells.

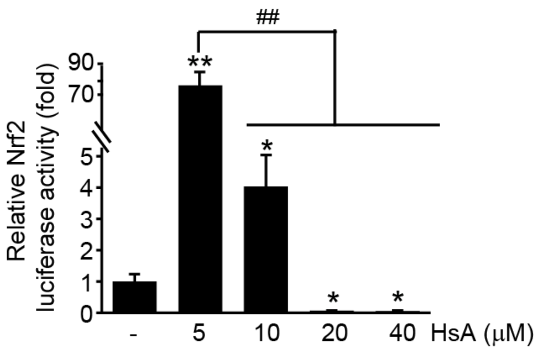

Figure S3. Nrf2 transactivation by HsA. Huh7 cells that had been transfected with pGL4.37[luc2P/ARE/Hygro] were treated with 5–40 μM HsA for 18 h. Luciferase activity in the cell lysates was normalized by protein concentration. $^{**}p < 0.01$, $^{*}p < 0.05$, significance versus vehicle-treated cells; $^{\#\#}p < 0.01$, significance versus 5 μM HsA-treated cells; Nrf2, nuclear factor erythroid 2-related factor 2.

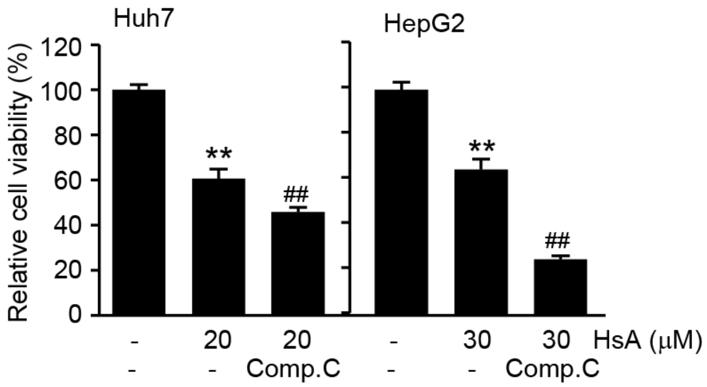

Figure S4. Effect of compound C on HsA-mediated toxicity. Huh7 (left) and HepG2 cells (right) were pre-incubated with 10 μM compound C for 1 h and then exposed to 20 or 30 μM HsA for 48 h. Relative cell viability was determined by MTT assay. $^{**}p < 0.01$, significance versus vehicle-treated cells; $^{\#\#}p < 0.01$, significance between HsA-treated cells; Comp. C, compound C.

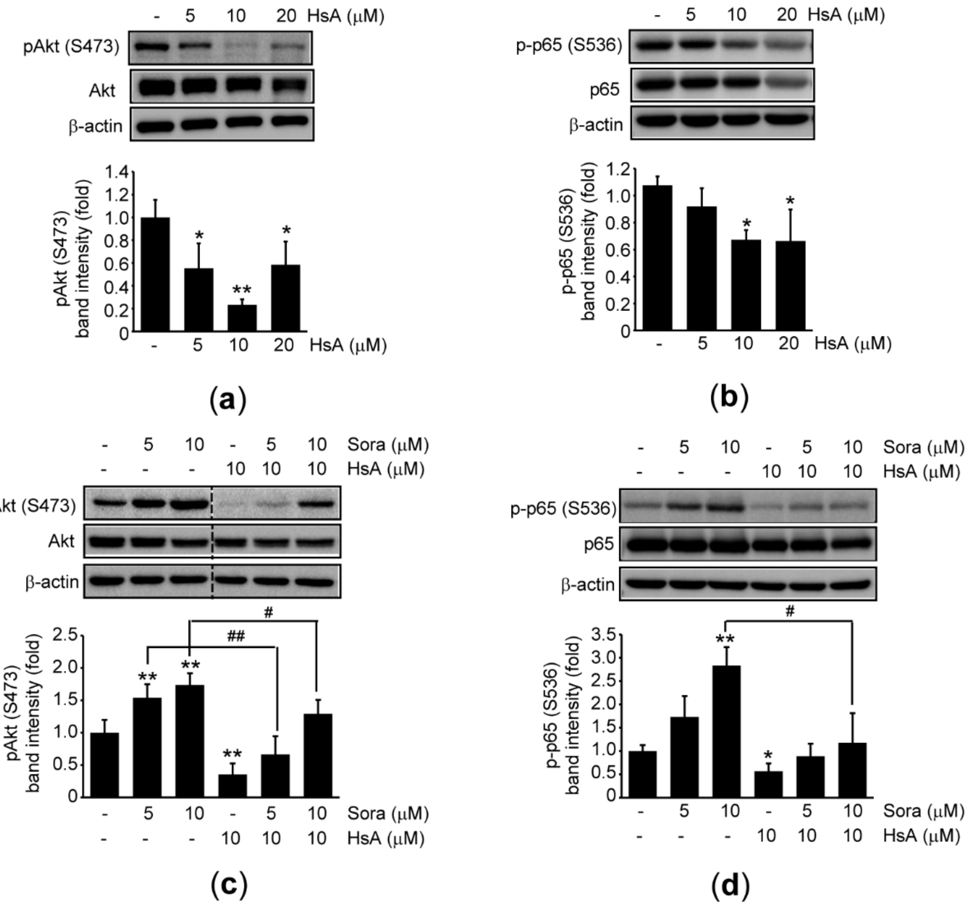

Figure S5. Effect of HsA on Akt and NF-κB phosphorylation in HCC cells. After Huh7 cells were exposed to 5-20 μM HsA alone (**a** and **b**) or 5-10 μM sorafenib in the presence 10 μM HsA (**c** and **d**) for 24 h, Akt and p65 phosphorylations were analyzed by immunoblotting (upper). Levels of phosphorylated Akt at S473 and phosphorylated p65 at S536 were quantified by densitometry (lower). Dashed lines in immunoblot of (**c**) indicate cropped images of the same membrane with the same exposure. ** $p < 0.01$, * $p < 0.05$, significance versus vehicle-treated cells; $^{\#\#}$ $p < 0.01$, $^{\#}$ $p < 0.05$, significance between sorafenib and sorafenib + HsA; pAkt, phosphorylated Akt; p-p65, phosphorylated p65; Sora, sorafenib.

Supplementary references

1. Kim, J.K.; Cho, I.J.; Kim, E.O.; Lee, D.G.; Jung, D.H.; Ki, S.H.; Ku, S.K.; Kim, S.C. Hemistepsin A inhibits T0901317-induced lipogenesis in the liver. *BMB Rep.* **2021**, *54*, 106–111. doi: 10.5483/BMBRep.2021.54.2.111.
2. Livak, K.J.; Schmittgen, T.D. Analysis of relative gene expression data using real-time quantitative PCR and the 2(-Delta Delta C(T)) Method. *Methods* **2001**, *25*, 402–408. doi: 10.1006/meth.2001.1262.
